# A cluster randomised controlled trial of an intervention to increase the implementation of school physical activity policies and guidelines: study protocol for the physically active children in education (PACE) study

**DOI:** 10.1186/s12889-019-6492-z

**Published:** 2019-02-11

**Authors:** Nicole Nathan, John Wiggers, Adrian E. Bauman, Chris Rissel, Andrew Searles, Penny Reeves, Christopher Oldmeadow, Patti-Jean Naylor, Angie L. Cradock, Rachel Sutherland, Karen Gillham, Bernadette Duggan, Sally Chad, Nicole McCarthy, Matthew Pettett, Rebecca Jackson, Kathryn Reilly, Vanessa Herrmann, Kirsty Hope, Adam Shoesmith, Luke Wolfenden

**Affiliations:** 1 Hunter New England Population Health, Hunter New England Area Health Service, Newcastle, Australia; 2 School of Medicine and Public Health, The University of Newcastle, Newcastle, NSW Australia; 3 Priority Research Centre for Health Behaviour, The University of Newcastle, Newcastle, NSW Australia; 4 Hunter Medical Research Institute, New Lambton Heights, NSW Australia; 5 Prevention Research Collaboration, Sydney School of Public Health, Sydney, Australia; 6 Charles Perkins Centre (D17), The University of Sydney, Sydney, NSW 2006 Australia; 7 School of Medicine, The University of Sydney, Camperdown, New South Wales Australia; 8 New South Wales Office of Preventive Health, Liverpool, New South Wales Australia; 9 School of Exercise Science, Physical and Health Education, University of Victoria, Victoria, British Columbia Canada; 10 Department of Social and Behavioral Sciences, Harvard T.H. Chan School of Public Health, Boston, MA USA; 11 Catholic Schools Office Diocese of Maitland-Newcastle, Newcastle, NSW Australia; 12 The NSW Department of Education, Newcastle, NSW Australia

**Keywords:** Physical activity, Policy, Schools, Implementation, Guidelines

## Abstract

**Background:**

In an attempt to improve children’s physical activity levels governments have introduced policies specifying the minimum time schools are to schedule physical activity each week. Despite this, the majority of schools in many jurisdictions fail to implement these policies. This study will assess the effectiveness of a multi-component implementation strategy on increasing the minutes of planned physical activity scheduled by primary school teachers each week.

**Methods:**

A cluster randomised controlled trial will be conducted in 62 primary schools in the Hunter New England region of New South Wales, Australia. Schools will be randomly allocated to receive either a multi-component implementation strategy that includes; obtaining executive support, training in-school champions, provision of tools and resources, implementation prompts, reminders and feedback; or usual practice. The study will employ an effectiveness-implementation hybrid design, assessing both policy implementation and individual (student) behavioural outcomes. The primary trial outcome of mean minutes of physical activity scheduled by classroom teachers across the school week will be measured via teacher log-book at baseline and approximately 12 and 18 months post baseline. A nested evaluation of the impact of policy implementation on child physical activity will be undertaken of students in Grades 2 and 3. Analyses will be performed using an intention to treat framework. Linear mixed effects regression models will be used to assess intervention effects on the primary outcome at both follow-up periods.

**Discussion:**

This study will be the one of the first well powered randomised trials internationally to examine the impact of an implementation strategy for a physical activity policy in primary schools and will address a fundamental research translation gap. Given the dearth of research, the findings will be important in informing future implementation efforts in this setting.

**Trial registration:**

ANZCTR ACTRN12617001265369 version 1 registered 1st September 2017.

## Background

Physical inactivity is the fourth leading cause of death worldwide [1] and is estimated to be responsible for approximately 6–10% of all non-communicable deaths or 5·7 million deaths globally [1]. International physical activity guidelines recommend that children aged 5–17 years accumulate at least 60 min of moderate to vigorous physical activity (MVPA) each day [2]. However, data from the United States (U.S.), United Kingdom (U.K.) and Australia suggest only a third of primary school aged children meet these guidelines [3–5]. As child physical activity patterns track into adulthood [4, 6], ensuring children are sufficiently physically active has been identified as a public health priority [7].

Schools are a key setting for the promotion of physical activity in children [6] as they provide convenient access to the majority of young people and possess the necessary facilities, personnel and ethos to engage children in activity [6]. Furthermore systematic reviews have found that interventions that increase opportunities to be physically active during the school day through regular quality physical education (PE), sport or physical activity in the classroom are effective in increasing children’s MVPA [8]. For example, reviews of school sport [9] and other structured activities in class such as energisers [10] have been shown to provide students with potentially 30mins of activity per day. As such, many governments have released guidelines or policy mandating minimum accumulated periods each week that primary schools are to schedule structured activity for children [11–14].

Despite the benefits of implementing such policies, research suggests that internationally most schools fail to implement physical activity policies at scale. For example, the 2014 physical activity report card for Ireland found that, based upon an audit of timetabled weekly PE of 419 schools, only 17% were providing the compulsory 2 h of PE per week [13]. Similarly, a 2011 U.S. study that undertook observations of 154 PE lessons found that only 5% of schools were compliant with mandated state policies that require 100 min of PE be taught each week [15]. A 2011 study found that only 43% of Canadian primary school teachers reported implementing the mandatory daily 30-min physical activity policy [14]. Furthermore a 2013 study, using 64 independent observers placed within Australian primary school classes for 9 weeks found only 13% of classes routinely engaged in physical activity during class time [16].

A recently published systematic review [17] of 17 qualitative and quantitative studies identified that primary schools face a number of barriers to the implementation of planned physical activities which relate to ‘environmental context and resources’ (e.g., availability of equipment, time or staff), ‘goals’ (e.g., the perceived priority of the policy in the school), ‘social influences’ (e.g., support from school boards), and ‘skills’ (e.g., teachers’ ability to implement the policy). Without the provision of implementation support to schools to overcome these barriers, the potential benefits of school-based physical activity policies on children’s health will not be realised [18]. However, there is little evidence regarding the most effective strategies to overcome these barriers and enhance implementation of physical activity policies in schools. A recent Cochrane review of the impact of implementation interventions in schools identified only one controlled trial in primary schools of a strategy to support the implementation of school physical activity policies [19]. The randomised trial undertaken in seven U.S. schools in 1994 aimed to enhance the quantity and quality of PE lessons by comparing the training of classroom teachers (whom received on-site training, intensive on-going technical assistance, modelling, audit and feedback, resources and coalition building support) to specialist PE teachers to control to improve teaching practices in PE lessons. Based on observational data of PE lessons, the study reported a significant improvement in implementation compared to control during the 3-year intervention period, however this was not sustained once the intensive support was removed.

The lack of evidence of effective strategies and their relative cost to support the implementation of physical activity policies represents a significant impediment to translation. Therefore, the primary aim of this trial is to assess the effectiveness and cost-effectiveness of a multi-component implementation strategy in increasing the minutes of planned weekly physical activity scheduled by classroom teachers consistent with the New South Wales (NSW) Government School Sport and Physical Activity Policy. As a secondary outcome of the trial, the study will assess the effectiveness of scheduled physical activity on children’s activity levels.

## Methods

The study methods will be reported in accordance with the CONSORT statement for cluster randomised controlled trials [20] and the Standards for Reporting Implementation Studies (StaRI) statement [21].

### Context

In 2015 the NSW Department of Education (DoE) amended its Sport and Physical Activity Policy (here after “policy”) [11], requiring students from Kindergarten to Year 10 to participate in a minimum of 150 min (increased from 120 min) of planned moderate with some vigorous physical activity across the school week. Planned physical activity includes time spent in PE, sport and other structured activities that is inclusive of all children and is part of their regular programming and planning. To support primary school teachers meet the policy requirements, the research team will target increases in planned physical activity across the three areas identified by the policy, that is; i) ***PE-*** teachers will be supported to programme PE throughout the school week by developing a scope and sequence for each school stage (K-2; 3–4; 5–6) and deliver active, effective and enjoyable PE. ii) ***Sport-*** teachers will be supported to programme sufficient time for sport and maximise student activity via strategies to improve student participation and enjoyment. iii) ***Other structured activities-*** teachers will be supported to integrate short-bouts of activity into class routines e.g. energisers [10] or active lessons [22]. Energisers are short classroom-based physical activities that break-up sitting time by getting students to engage in short bursts i.e. 3–5 min of MVPA involving no equipment. Active lessons integrate physical activity into another subject area by making the traditional lesson more active, for example, getting students to skip as they recite their times tables.

### Design and setting

**Fig. 1:**
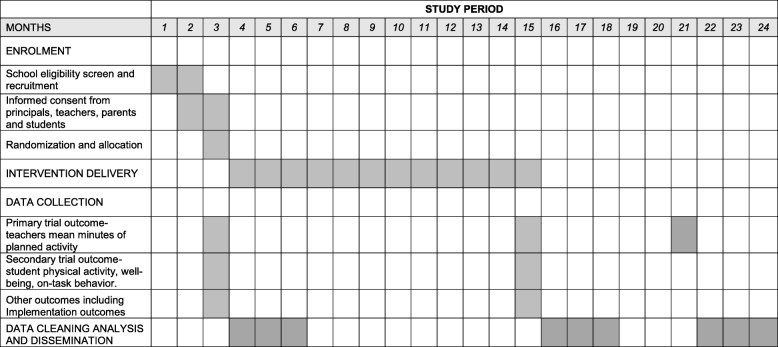
Time schedule of participant enrolment, data collection and intervention delivery

This study will employ a cluster randomised controlled trial (RCT). Sixty-two primary schools, in the Hunter New England (HNE) region, of NSW will be randomised to receive either a multi-component implementation strategy to support policy implementation or ‘usual practice’. The trial will assess between-group differences in the mean minutes of scheduled weekly physical activity with data collected at baseline (Oct 2017- Feb 2018), and immediately following the delivery of the implementation strategy (Oct-Dec 2018). To determine the longer-term sustainability data will be collected approximately 6-months following completion of the implementation strategy (April–June 2019) (see Fig. 1).

### Participants and recruitment

#### Schools

All government and Catholic schools in the study region (approximately 90% of all schools) will serve as the sampling frame. Schools participating in another physical activity intervention, schools with both primary and secondary students (i.e. central schools) and schools catering exclusively for children with special needs will be excluded. School principals will be provided with a study information package and asked to provide written informed consent. Recruitment will continue until the sample of schools have consented.

#### Teachers

Following principal consent, a member of the research team will attend a school staff meeting to provide teachers with a brief overview of the purpose of the study and to answer any questions teachers may have. Consenting teachers will be invited to complete a paper survey during the week of their school’s scheduled data collection visit which will include a log-book of their class scheduled physical activity.

#### Students

As the effect of scheduling physical activity on children’s physical activity has been established in a previous pilot of this study [23] for pragmatic reasons only a subset of school students i.e. those in grades 2 and 3 will be invited to take part in the accelerometer data collection component of the trial. An information package will be sent to parents of students in participating schools encouraging them to discuss the study procedures with their child and to invite study participation. Two weeks following distribution of the information packages, parents who have not returned a consent form will be telephoned by staff employed through the school and asked if they would like to consent to child participation.

#### Parents

Parents of students in grades 2 and 3 who consent to have their child participate in data collection will be invited to complete a telephone survey regarding the physical activity and wellbeing of their children. Parents who are interested in completing the survey will be asked to include their telephone number on their child’s consent form.

### Randomisation and blinding

Following baseline data collection, an independent statistician will set-up block randomisation using a computerised random number function to randomise schools in a 1:1 ratio to either an intervention or control group. Block randomisation will ensure group allocation is approximately equal. Allocation will be stratified by the geographic (rural vs urban) location of the school given the association with implementation of school physical activity policies or practices [24]. Allocation will follow baseline data collection. Due to the nature of the intervention school staff will be aware of school group allocation. Whilst all efforts will be made to keep data collectors blinded to group allocation, due to the provision of some resources to schools (e.g. manuals) they may become aware of group allocation during attendance at the school for follow-up data collection. Data entry staff will be blinded.

### Intervention group: implementation strategy

#### Development and theoretical framework

The implementation strategy has been developed using both the Behaviour Change Wheel (BCW) and Theoretical Domains Framework (TDF) [25], which together ensure; a comprehensive assessment of factors (i.e. capabilities, opportunities and motivation) impacting on an individual’s behaviour are considered, and that modifiable factors and potential behaviour change techniques that may be utilised to influence the desired behaviour of an individual are identified. Following extensive formative research which included i) literature reviews; ii) interviews using an adapted form of the validated TDF survey with 76 primary school teachers and iii) observations of teachers’ delivery of PE, sport and the school environment, the recommended process described by Michie et al. [26] was undertaken to map the identified barriers to the BCW and TDF. Potential behaviour change techniques and implementation strategies were then identified. Following consultation with an advisory group consisting of, implementation and health behaviour scientists, physical activity experts, teachers, principals and senior government policy makers (who will oversee delivery of the study) the proposed implementation strategies were presented and discussed. To be included, implementation strategies were also assessed against the APEASE criteria [25], a systematic approach for considering contextual factors during the selection of implementation strategies, which includes; Affordability (can be delivered on budget), Practicality (is feasible to deliver), Effectiveness and cost-effectiveness (it works), Acceptability (to the school community), Side-effects/safety (no negative consequences), Equity (no groups disadvantaged in particular Aboriginal or Torres Strait Islander communities). The selected implementation strategies have previously been utilised by members of the research team to successfully change the health promoting policies and practices of schools [27, 28] and other organisations [29–33]. Table 1 describes each of the implementation strategies using the Expert Recommendations for Implementing Change (ERIC) taxonomy [34] and shows how these were mapped against the BCW and TDF to address barriers to practice change.

**Table 1:** Description of implementation strategies mapped to the relevant theories and taxonomies

Implementation strategy	Proposed mechanism of action			Intervention content	
	Barriers addressed	COM-B and (TDF)	Intervention functions	BCT Behaviours	Detailed explanation
Centralize technical assistance and Provide ongoing consultation	Teachers knowledge, ability or competence Lack of time Perceived priority of the policy in the schools	Psychological capability (beliefs about capabilities; knowledge) Opportunity- social (environmental context and resources) Motivation- reflective (goals)	Enablement Persuasion	Review behaviour goal(s) Review outcome goal(s)	Project officers (a PE teacher and health promotion practitioner) will provide technical assistance to schools throughout the study period, to support policy implementation by working directly with schools and school champions to overcome barriers and provide expertise support and resources. Project officers will provide ongoing consultation to in-school champions via telephone, email or if needed face-to face to support implementing the intervention. The focus of these meetings will be to support school champions brain storm solutions to barriers as they arise, review progress of the schools implementation plan and if necessary modification and re-setting of goals.
Mandate change	Support from school boards Physical activity considered a lower priority than other subjects	Opportunity- Social (Social influences) Motivation- reflective (Goals)	Enablement	Commitment	Project officers will meet face to face with principals and school executives to communicate the importance and benefits of policy implementation. The school executive will be asked to demonstrate support for the implementation of the policy through the development of a “Sport and Physical Activity Procedures document” (as required by the policy) and to communicate (e.g. via newsletters, assemblies and staff meetings) expectations to staff, students and parents regarding changes to programming of PE, sport and other activities.
Identify and prepare champions	Lack of time in the curriculum Teachers knowledge, ability or competence	Opportunity- social (environmental context and resources) Psychological and physical capability (beliefs about capabilities)	Modelling; Education; Training	Identification of self as role model Social support (unspecified) Problem solving Instruction on how to perform a behaviour Demonstration of the behaviour	Each school will nominate up to three in-school champions (existing teachers at the school) who will drive implementation of the intervention in their school and with support from project officers overcome indifference/ resistance that the intervention may provoke in the school. They will be encouraged to role model the desired behaviours as it will be an example to other teachers. To prepare in-school champions for their role they will complete a 1-day (5-h) face-to-face workshop run by project officers which will include; education about the policy, instruction and demonstration of physical activity energisers and PE lessons and time to begin action planning which will require in-school champions to identify barriers/ facilitators, to implementation and possible solutions to overcome these via a “if-then-what” plan. The training has been accredited by the state educational authority and provides time towards teachers continuing professional development hours.
Develop a formal implementation blueprint.	Perceived priority of the policy in the schools	Motivation- reflective (Goals)		Goal setting (behaviour) Action planning Graded tasks	School champions will be supported to develop a plan for the implementation of the policy in their school. The plan will identify what the school is aiming to specifically achieve, the strategies to do so and by when, the resources available or required to implement the plan. The plan will be broken into school terms to allow school champions to break up some of the more complex policy requirements into achievable tasks.
Conduct educational outreach visits.	Teachers knowledge, ability or competence	Psychological and physical capability (beliefs about capabilities)	Education; Training	Instruction on how to perform a behaviour Demonstration of the behaviour Instruction on how to perform a behaviour Framing/ reframing Verbal persuasion about capability	Project officers will meet with all teachers (face to face) as a group in each school for 1-2 h to; • Introduce the in-school champion and their role in implementing the intervention and as a point of support in the school; • Educate teachers about the policy with a deliberate aim to reframe policy adoption from “adding to teacher load” but rather easily integrated into existing routines. • Provide verbal persuasion about the teachers capability to implement the policy; • Instruct and demonstrate physical activity energisers and PE lessons; • Prompt habit formation for some of the physical activity practices;
Develop and distribute educational materials	Teachers knowledge, ability or competence	Psychological capability (beliefs about capabilities; knowledge)	Education; Training	Adding objects to the environment	In-school champions will receive an “intervention manual” which will include policy and timetable templates, exemplar physical activity timetables and physical education curriculum schedules. Classroom teachers will receive various educational materials including practical games and strategies for increasing physical activity in lessons. These materials will be available in print and via an online portal. The portal will also contain professional learning videos for all teachers (including school champions) which reinforces the information they have received via face to face training.
Capture and share local knowledge	Teachers knowledge, ability or competence Lack of time in the curriculum	Opportunity- social (social influences) Motivation-reflective (belief about consequences)	Modelling; Persuasion	Social comparison	Project officers will develop “case studies” from other intervention schools on how school champions and teachers made “something work” in their setting. This will be utilised during project officers ongoing consultation meetings with in-school champions and included on the online portal as an “infocus school”.
Change physical structure and equipment	Availability of equipment	Opportunity- physical (environmental context and resources)	Environmental restructuring	Restructuring the physical environment	School champions will be encouraged to develop “physical activity packs” for all teachers to keep in each classroom which includes a class set of basic physical activity equipment e.g. bean bags, balls, hoops etc. from the schools’ existing sports equipment enabling teachers to implement integrated physical activity lessons and PE lessons easily.

### Data collection and measures

#### Primary trial outcome- mean minutes of planned weekly physical activity scheduled by classroom teachers

The primary trial outcome is the mean minutes of physical activity scheduled during a 1-week data collection periods at baseline, 12 and 18 months following baseline. Scheduled physical activity includes time spent in PE, sport and other structured physical activities - as required to be compliant with the DoE Sport and Physical Activity Policy. Time scheduled for physical activity for each class will be assessed via class teacher completion of a daily activity log-book, which has been previously utilised by the project team [23]. At the end of each day of the week of data collection, each teacher responsible for the class that day will complete a written log of the day’s teaching including the time and occasions of physical activity for PE, sport or other structured activities i.e. energisers or active lessons. The use of teacher log-books is frequently used in classroom-based obesity prevention interventions [35, 36] with high response rates (i.e. > 80%) [35] and established reliability and validity [37].

#### Secondary outcomes

##### Mean minutes of planned PE, sport and other structured activities scheduled by classroom teachers

as the policy allows physical activity to be accrued via PE, sport and other structured activities such as energisers and active lessons, the mean time classes participate in each of these activities will be collected from teacher log-books (as per the primary outcome) at baseline, 12 and 18 months following baseline. The mean difference between groups will be compared to identify what the intervention was most able to influence.

##### Student physical activity and sedentary behaviour

Students’ school-day physical activity will be assessed at baseline and 12-month follow-up via an ActiGraph GT3X+ accelerometer (ActiGraph Corporation, Pensacola, FL). Accelerometers have displayed acceptable intra-and inter-instrumental reliability and provide a valid and reliable estimate of physical activity in young people.32–34 Accelerometers will be attached to a watch band and students will be asked by trained research assistants to wear the accelerometers on their non-dominant hand from Monday through to Friday, during school hours only (i.e. 9 am-3 pm). Accelerometers will be worn by children as soon as they enter their class for the day and removed when the bell for finishing school rings. Teachers will be responsible for distributing and collecting the accelerometers on a daily basis. Students will be asked to wear the accelerometers for the whole school day except for water-based activities. Student data will be analysed if accelerometers are worn for ≥80% of the school day on ≥3 days. Accelerometer non-wear time will be calculated by summing the number of consecutive zero counts accumulated in strings ≥20 min. Wear time will be estimated by subtracting non-wear time from the total monitoring time for the school day. For each valid school day, counts per minute (cpm) will be calculated by dividing the total accelerometer counts by the minutes of wear time. Accelerometer counts will be classified as sedentary, light-intensity PA, and MVPA using the vertical axis wrist cut-points developed by Chandler et al. [38]

##### Student physical activity outside of school hours

whilst the purpose of the policy is to increase physical activity during school hours, to identify any compensatory physical activity behaviour occurring out of school hours [39] parents will be asked to report via the telephone survey, at baseline and follow-up, on their child’s physical activity outside of school hours and on weekends. Measures will be taken from the 2011–2012 NSW child population health survey [40].

##### Student well-being

previous research indicates quality of life is associated with increased physical activity among children [2]. To further assess the impact of the intervention, the differences between groups at follow-up in Pediatric Quality of Life Inventory as reported by parents via the telephone survey will be assessed as a secondary outcome of the trial.

##### Student on-task behaviour

breaking up long periods of sitting time with physical activity is associated with increased attention and focus of children [10]. At baseline and follow-up teachers will, as part of their paper survey, be asked to complete selected items from the Teaching and Learning International Survey (TALIS) (OECD 2010), which will provide a class-based measure of student’s on-task behaviour.

#### Implementation outcomes

To characterise implementation the measures recommended by Proctor et al. [41] of implementation outcomes will also be assessed. This includes;

- Acceptability- defined as *the perception among implementation stakeholders that a given treatment, service, practice, or innovation is agreeable, palatable, or satisfactory.* At follow-up intervention principals and teachers will be asked to complete, via paper based survey, the Acceptability of Intervention Measure (AIM) [42], developed by Weiner et al., a four-item valid and reliable scale.

- Adoption- defined as *the intention, initial decision, or action to try or employ an innovation or evidence-based practice.* Based upon a previously developed tool from the research team [43] at baseline and follow-up all intervention and control principals will be asked to report, via paper based survey, their stage of adoption for implementing the physical activity policy.
- Appropriateness- defined as *the perceived fit, relevance, or compatibility of the innovation or evidence based practice for a given practice setting, provider, or consumer; and/or perceived fit of the innovation to address a particular issue or problem.* At follow-up intervention principals and teachers will be asked to complete, via paper based survey, the Intervention Appropriateness Measure (IAM), a four-item valid and reliable scale.
- Feasibility- defined as *the extent to which a new treatment, or an innovation, can be successfully used or carried out within a given agency or setting.* At follow-up intervention principals and teachers will be asked to complete, via paper based survey, the Feasibility of Intervention Measure (FIM), a four-item valid and reliable scale.
- Fidelity- defined as *the degree to which an intervention was implemented as it was prescribed in the original protocol or as it was intended by the programme developers*. Project records as well as post-intervention questionnaires completed by intervention principals, school champions and teachers will be used to determine the proportion of schools that received and attended to each of the implementation strategies.
- Implementation cost- defined as *the cost impact of an implementation effort;* see cost and cost-effectiveness measure below.
- Penetration- defined as *the integration of a practice within a service setting and its subsystems* will be measured as per the primary trial outcome to assess the proportion of teachers scheduling the required minutes as per the DoE Sport and Physical Activity Policy. Penetration will then be calculated by the number of teachers who meet the policy requirements, divided by the total number of teachers expected to implement the policy.
- Sustainability- defined as *the extent to which a newly implemented treatment is maintained or institutionalized within a service setting’s ongoing, stable operations* will be measured as per the primary trial outcome approximately 6 months following the completion of the implementation strategy.

#### Other measures

##### School characteristics

Data regarding the operational characteristics of schools, school participation in other physical activity programmes and implementation activity will be collected during a survey of school principals and classroom teachers. The baseline characteristics of those who have complete primary outcome data will be compared with those who dropped out from the study in order to investigate differences between them. Items will be sourced from previous surveys of school principals conducted by the research team [44, 45], which have achieved participation rates of between 70 and 96% [44].

##### Intervention cost and cost effectiveness

The costs and resource use associated with the intervention will be collected prospectively from project records (staff and consumables), teacher surveys and records of the School Sport Unit. Costs will be categorised as implementation strategy development, execution or maintenance. Additional costs in the intervention group are anticipated to be labour (policy implementation support); programme development and training costs; and resource costs (materials). Where data are unavailable, the basis for cost modelling assumptions will be detailed. Subject to assessment of effectiveness, a trial-based cost effectiveness analysis (CEA) will be conducted from multiple stakeholder perspectives. The reportable outcomes will be average cost-effectiveness and incremental cost effectiveness ratios. Sensitivity and scenario analyses will be undertaken to test the impact of changing key design features of the intervention and scale-up of the implementation model.

#### Overall data management

Management of trial data will be in accordance with a data management protocol, which has been developed and approved by the project’s advisory group. Data will be stored securely as per the requirements of the Hunter New England Human Research Ethics Committee and The University of Newcastle Human Research Ethics Committee. Data will only be accessible to primary researchers and statisticians. Confidential participant data will be stored securely and not linked to survey responses.

#### Analysis and sample size

Analyses will be performed under an intention to treat framework, with the class (nested within a school) the unit of analysis. Separate analyses will be performed at each follow-up time point. Intervention effects on the primary trial outcome (at each follow-up time point) will be assessed using a linear mixed effects regression model, which will include fixed effects for treatment group (intervention vs control), the baseline value of the outcome and variables that are prognostic of the outcome (geographic and socio-economic location of the school) [24]. We will include a random effect for the school to allow for the clustering of classes within schools. Multiple imputations will be performed as part of a sensitivity analysis for schools not providing follow up data in accordance with the recommendation by White et al. [46] The continuous secondary outcomes will be analysed using a linear mixed effects regression model, with fixed and random effects as outlined for the primary outcome. Student level outcomes will include an additional random effect for class (nested within school) and allow for repeated measures at different follow-up time points through a compound symmetric residual correlation matrix. Based on data held by the research team, the average primary school in the study region will have 13 classrooms. Using a conservative estimate of a 70% response rate from classrooms teachers and assuming 20% loss-to-follow-up, a sample of 31 intervention and 31 control schools will provide a sample of approximately 450 classes (225 intervention and 225 control) at follow-up. Assuming a standard deviation of 45mins at follow-up in the comparison group, and a conservative intra class correlation coefficient of 0.2 the sample will be sufficient to detect an absolute difference of 18.0 min, with 80% power and an alpha of 0.05.

#### Control group and contamination

The delivery of all intervention components, including communication strategies will be under the control of the research team, and will not be provided to comparison group schools during the intervention period. Schools in the control group will receive ‘usual’ implementation support. Implementation support provided to schools as part of policy dissemination involves the provision of information and resources via a website, including factsheets, example policies and templates. According to evidence [47] and theory [48] such strategies do not address the primary impediments to policy implementation and that any impact of such initiatives on the primary trial outcome is likely to be minimal. Nonetheless, data regarding schools’ exposure to such support and other potential sources of contamination will be assessed.

#### Research trial governance

This study has employed a research co-production approach in its design [49]. Similarly an advisory group consisting of researchers, policy makers, practitioners and experts within the school and health setting will oversee all aspects of the planning, implementation and evaluation of the project. A project team consisting of research staff and practitioners will develop and operationalise implementation strategies and data collection components of the trial according to study protocol. The advisory group will oversee the project dissemination plan including all publications and reports to stakeholders. Authorship will conform to the International Committee of Medical Journal Editors (ICMJE) guidelines.

#### Trial discontinuation or modification

It is not anticipated that any events would occur that would warrant discontinuing the trial. Any unforeseen adverse events will be reported to the Hunter New England Human Research Ethics Committee (primary approval committee) and advice sought regarding required action. The trial registration record will be updated with any protocol modifications and any deviations from original protocol will be reported in study outcome papers.

## Discussion

Australian physical activity guidelines recommend children accumulate at least 60 min of moderate to vigorous physical activity (MVPA) each day [8]. However, similar to other countries [4], statistics indicate only 1 in 4 (23%) of Australian primary school children meet the recommended level of physical activity [3]. Improvements in scheduled physical activity in schools has the potential to increase child physical activity and hence should be embedded into a schools culture to maximise public health benefits.

This protocol comprehensively outlines the methodology to be undertaken to assess the effectiveness of a multi-component implementation support strategy to improve the implementation of the NSW Sport and Physical Activity Policy of 150 min of scheduled physical activity during a school week. Given the limited evidence base the trial will provide rigorous and theory informed methods to generate new knowledge regarding implementation of evidence-based policies and guidelines in schools.

## Abbreviations

BCW: Behaviour Change Wheel
DoE: Department of Education
MVPA: Moderate to vigorous physical activity
NSW: New South Wales
PE: Physical Education
TDF: Theoretical Domains Framework
